# Efficient methylene blue elimination from water media by nanocomposite adsorbent-based carboxymethyl cellulose-grafted poly(acrylamide)/magnetic biochar decorated with ZIF-67

**DOI:** 10.1039/d5ra03796d

**Published:** 2025-09-08

**Authors:** Seyed Jamaleddin Peighambardoust, Somayyeh Rezaei-Aghdam, Javaneh Sakhaei Niroumand, Parisa Mohammadzadeh Pakdel, Mika Sillanpää

**Affiliations:** a Faculty of Chemical and Petroleum Engineering, University of Tabriz Tabriz 5166616471 Iran j.peighambardoust@tabrizu.ac.ir; b Saveetha School of Engineering, Saveetha Institute of Medical and Technical Sciences, Saveetha University Chennai Tamil Nadu 602105 India; c Department of Civil Engineering, University Centre for Research & Development, Chandigarh University Gharuan, Mohali Punjab India; d Institute for Nanotechnology and Water Sustainability (iNanoWS), Florida Campus, College of Science, Engineering and Technology, University of South Africa Johannesburg 1710 South Africa

## Abstract

A free radical polymerization approach was applied to synthesize different carboxymethyl cellulose-grafted poly(acrylamide) hydrogels (Hyd) composited with biochar, magnetic biochar, and magnetic biochar decorated with ZIF-67 to decontaminate methylene blue (MB) from water media. Biochar was obtained from walnut shells (WS) by a pyrolysis method, and magnetic biochar (WS/CoFe_2_O_4_) and biochar-decorated ZIF-67 (WS/CoFe_2_O_4_/ZIF-67) were prepared by chemical co-precipitation and hydrothermal methods, respectively. An increase in the amount of these particles by up to 10 wt% enhanced the removal performance. The maximum removal performance by Hyd, Hyd/WS, Hyd/WS/CoFe_2_O_4_, and Hyd/WS/CoFe_2_O_4_/ZIF-67 was computed to be 85.74%, 91.74%, 95.83%, and 97.72%, respectively, at optimum conditions of pH = 10, adsorbent dose = 1 g L^−1^, contact time = 50 min, initial concentration = 10 mg L^−1^, and temperature = 25 °C. Pseudo-second-order kinetic and the Freundlich isotherm models had the highest desirability in the kinetic and equilibrium data regression, respectively. The thermodynamic study showed the spontaneity and exothermic nature of the MB decontamination process. Ultimately, the synthesized adsorbents, specifically Hyd/WS/CoFe_2_O_4_/ZIF-67, could be used for wastewater treatment.

## Abstract 2

Scheme of MB adsorption mechanism by Hyd/WS/CoFe_2_O_4_/ZIF-67 nanocomposite adsorbent.

## Graphical abstract

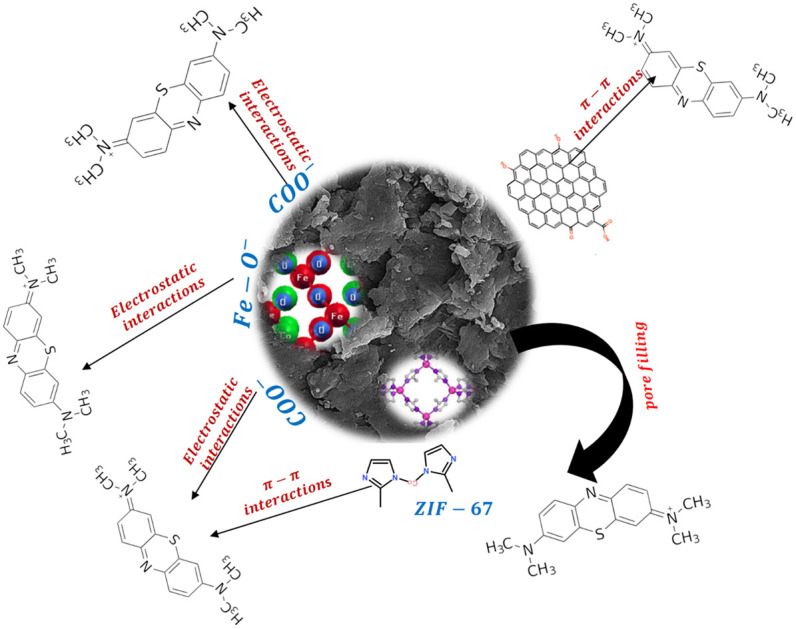

## 1. Introduction

Water is the main substance required by living organisms. Water resources cover about 70% of the Earth's surface, while only 0.002% is available for human consumption. Surface water and groundwater are used extensively in agriculture, power generation, livestock production, industrial activities, forestry, fisheries, shipping, and recreational activities. According to the United Nations reports, the demand for freshwater has increased significantly due to the rapid population growth and industrial consumption.

Toxic chemicals, industrial or domestic wastewater, agricultural chemicals, and organic and inorganic materials can be among the most critical pollutants of water resources. Organic pollutants that can contaminate water resources include antibiotics and synthetic dyes. Organic pollutants such as dyes are widely used in the textile, paper, cosmetic, pharmaceutical, paint, leather, food, and plastic industries, with textile wastewater discharge accounting for 15% of the total production of dyes worldwide. Toxic chemicals, industrial or domestic wastewater, agricultural chemicals, and organic and inorganic materials can be among the most critical pollutants of water resources.^1^ In addition, even small amounts of these dyes in water (less than 1 ppm) can limit the penetration of sunlight, damaging the photosynthesis process in aquatic plants.^2^

Methylene blue (MB), as a water-soluble cationic dye, is extensively utilized in industry for dyeing leather and cotton and for printing and pharmaceutical purposes. With the development of the printing and dyeing industries, large amounts of MB are released into the aquatic environments through wastewater. Its entry into the human body can cause shock, profuse sweating, vomiting, increased heart rate, cyanosis, jaundice, mental confusion, and eye and skin irritations.^3^

Various approaches can be used to remediate wastewater from these industries, including membrane separation,^4^ adsorption,^5^ chemical oxidation,^6^ coagulation,^7^ and ion exchange.^8^ Adsorption is recognized as a promising technology for water remediation owing to various reasons such as its simple design, low cost, high removal efficiency, ease of operation, and availability compared with other water treatment techniques.^9^ Hydrogels are polymeric materials with a hydrophilic structure that can retain significant quantities of water within their three-dimensional structures without dissolving.^10^ Based on the nature of crosslinking, hydrogels are of two types, *i.e.*, physical hydrogels, in which the polymeric chains are joined by different physical bonds, including hydrogen bonding, crystallization, and hydrophobic interactions, and chemical hydrogels, where the polymeric chains are linked through covalent bonds.^11^ They can be used as adsorbents to eliminate dyes, heavy metals, and antibiotics from wastewater through various mechanisms such as hydrogen bonding, electrostatic interaction, and chelation. To promote the mechanical strength and removal efficiency, various materials can be embedded in the hydrogel matrix, such as clay-based materials,^12^ carbon-based materials,^13^ magnetic materials,^14^ and metal–organic frameworks (MOFs).^15^ Recently, biochar as an adsorbent produced from inexpensive, abundant, and readily available precursors such as agriculture waste has been widely applied.^16^ Among the various precursors based on agricultural waste, the chemical composition of walnut shells (WS) has shown great potential as a carbon source for producing biochar to remove target pollutants from aqueous solutions.

Magnetic nanoparticles have wide applications in the medical, electronics, environmental, and industrial sectors. These nanoparticles have great potential in degrading and removing pollutants such as pesticides, antibiotics, heavy metals, and dyes. Magnetic CoFe_2_O_4_ nanoparticles have been considered promising adsorbents for doping biochar due to their large specific surface area, outstanding chemical stability, easy separation by an external magnetic field, and high mechanical strength.^17^

Another approach to enhance the surface area and removal performance of magnetic biochar is its modification with MOFs. MOFs have attracted special attention for various applications due to their structural diversity, tunable structure, and ability to tune their pore size and porosity.^18^ Generally, MOFs are synthesized by bonding metal ions and ligands under specific conditions. Various methods have been used to synthesize MOFs, such as solvothermal, hydrothermal, mechanochemical, sonochemical, and sol–gel methods.^19^ Zeolitic imidazolate frameworks (ZIFs) are a class of MOFs known for their outstanding chemical and hydrothermal stability. They feature structures similar to zeolites and possess desirable characteristics, including a large surface area, porosity, and pore sizes.^20^ ZIF-67 is a family of ZIFs created by bonding 2-methyl imidazolate with cobalt ions, forming a three-dimensional porous structure with the shape of a rhombic dodecahedron.^20^ Recently, ZIF-67 nanoparticles have been widely applied in wastewater treatment due to their remarkable features, including high surface area and chemical and mechanical stability.

Magnetic biochar decorated with ZIF-67 was incorporated into a CMC-g-P(AAm) hydrogel as a new adsorbent for removing MB from water environment. FTIR, XRD, VSM, BET, and SEM-EDX approaches verified the successful synthesis of fillers and nanocomposite hydrogels. The filler content was optimized based on the swelling and removal performance. The influence of adsorption parameters, including pH, adsorbent dose, sorption time, initial MB concentration, and temperature, were assessed in detail. Also, studies of kinetic and isotherm models were performed using non-linear models. A thermodynamic study was also conducted to identify the nature of the adsorption process.

## 2. Materials and methods

### 2.1. Materials

Potassium persulfate (KPS, 270.32 g mol^−1^, 99.0%) and CMC were purchased from Samchun, Korea. Acrylamide (AAm, 71.08 g mol^−1^, 99.9%), methylene bisacrylamide (MBA, 154.17 g mol^−1^, 99.0%), cobalt nitrate hexahydrate (Co(NO_3_)_2_·6H_2_O, 291.03 g mol^−1^,≥99.0%), iron(iii) chloride hexahydrate (FeCl_3_·6H_2_O, 270.30 g mol^−1^, 99.9%), 2-methylimidazole (C_4_H_6_N_2_, 82.10 g mol^−1^, >98%), methanol (CH_3_OH, 32.04 g mol^−1^, ≥99.5%), sodium hydroxide (NaOH, 40 g mol^−1^, 99.0%), MB (319.85 g mol^−1^, 99.0%) and hydrochloric acid (HCl, 36.46 g mol^−1^, 38%) were bought from MERCK, Germany. Walnuts were purchased from a local shop, and deionized (DI) water was used throughout the experiments.

### 2.2. Synthesis of biochar (WS) and magnetic biochar (WS/CoFe_2_O_4_)

To eliminate impurities and dust, the walnut shells were rinsed several times with DI water and dried in an oven at 65 °C for 24 h. The dried walnut shells were carbonized in a furnace equipped with a nitrogen line at a temperature of 700 °C for 2 h. The obtained biochar was ground by a mill and sieved through meshes (ASTM E 11, 25 NUM). The magnetization of WS was performed using a chemical precipitation method. A specific amount of WS (1 g) was dispersed in 100 mL DI water. Then, 0.75 g of Co(NO_3_)_2_·6H_2_O and 1.45 g of FeCl_3_·6H_2_O were added to the WS suspension and mixed for 30 min. The temperature of the prepared suspension was set at 80 °C, and 50 mL of 3 M NaOH was added dropwise to reach a pH of 11. After mixing the dark suspension for 2 h, the magnetic WS was isolated by a magnet and washed with DI water several times. The WS/CoFe_2_O_4_ particles were dried in an oven for 24 h at a temperature of 80 °C.

### 2.3. Synthesis of ZIF-67-decorated WS/CoFe_2_O_4_, hydrogel, and nanocomposite hydrogel

**Fig. 1:**
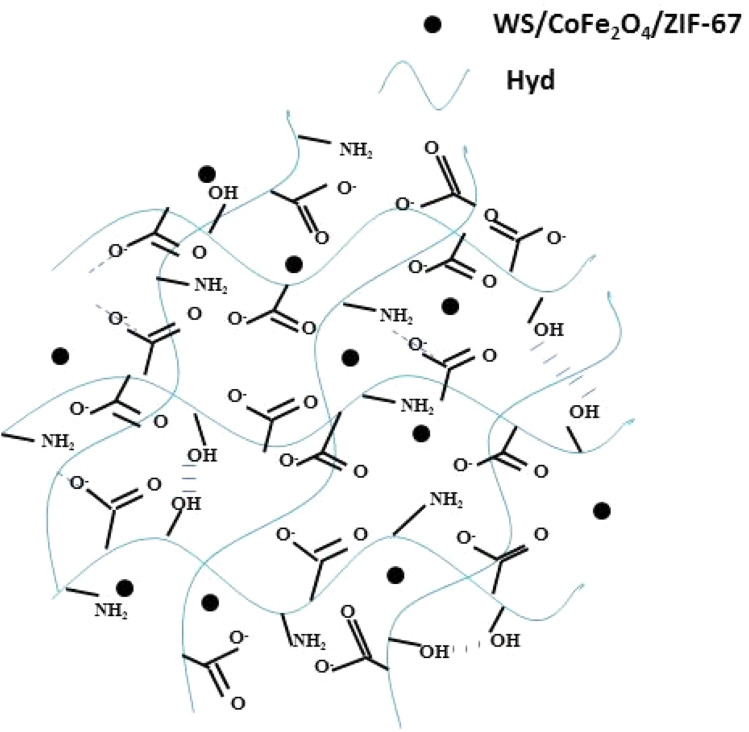
Scheme of Hyd/WS/CoFe_2_O_4_/ZF-67.

To prepare WS/CoFe_2_O_4_/ZIF-67, 0.7 g of Co(NO_3_)_2_·6H_2_O was dissolved in 50 mL of methanol at room temperature. Then, 1 g of WS/CoFe_2_O_4_ was added to this solution and stirred for 20 min. In another beaker, 1.6 g of 2-methylimidazole was dissolved in 10 mL of methanol and added slowly to the WS/CoFe_2_O_4_ suspension. The suspension was stirred for 24 h at room temperature using a magnetic stirrer. After ending the reaction time, the separated magnetic nanocomposite was washed with methanol several times and dried in a thermal oven for 24 h at 65 °C. The synthesis of the CMC-g-P(AAm) hydrogel (Hyd) *via* the free radical polymerization method was similar to our previous works, with the difference being that WS, WS/CoFe_2_O_4_, and WS/CoFe_2_O_4_/ZF-67 were used in the synthesis of the nanocomposite hydrogels.^21^ To begin the hydrogel synthesis, 0.33 g of carboxymethyl cellulose (CMC) was fully dissolved in deionized water at an elevated temperature. Separately, a dispersion containing 1.2 g of acrylamide (AAm), 0.3 g of potassium persulfate (KPS), 0.03 g of *N*,*N*′-methylene bisacrylamide (MBA), and a designated filler was prepared and subjected to ultrasonic treatment for 30 min to ensure uniformity. This homogenized mixture was introduced into the cooled CMC solution, followed by nitrogen gas purging for 5 min to eliminate dissolved oxygen. The resulting mixture was transferred to a reaction vessel and maintained in a 65 °C water bath. Gelation occurred within 5 min, after which the system was held at the same temperature for another 2 h to complete the free radical polymerization process. The resulting hydrogel nanocomposite was sliced into smaller fragments and repeatedly rinsed with deionized water to eliminate residual reactants. Finally, the hydrogel pieces were oven-dried at 60 °C for 24 h, pulverized using a mechanical grinder, and passed through a 40–60 mesh sieve to obtain a uniform particle size. The scheme of Hyd/WS/CoFe_2_O_4_/ZF-67 is presented in Fig. 1.

### 2.4. Characterization

The functional groups and chemical structure of the prepared samples were identified using FTIR spectroscopy (Tensor 27, Bruker, Germany) operating in the wavenumber range of 400 to 4000 cm^−1^. The X-ray diffraction (XRD) patterns of WS, WS/CoFe_2_O_4_, Hyd, Hyd/WS, Hyd/WS/CoFe_2_O_4_, and Hyd/WS/CoFe_2_O_4_/ZIF-67 adsorbents were recorded using an X-ray diffractometer (Krisallofex D500, Siemens, Germany) equipped with Cu Kα (*λ* = 1.54 Å) radiation. The surface morphology of WS, WS/CoFe_2_O_4_, Hyd, Hyd/WS, Hyd/WS/CoFe_2_O_4_, and Hyd/WS/CoFe_2_O_4_/ZIF-67 adsorbents was analyzed by scanning electron microscopy (SEM, MIRA3, TESCAN, Brno, Czech Republic) operating at a voltage of 15 kV. The specific surface area, mean pore diameter and total pore volume of the samples were calculated using an N_2_ gas sorption instrument (Asap 2020, Micro-metrics, USA).

### 2.5. Swelling study

0.1 g of adsorbent (*m*_i_) was placed in 200 mL DI water for the swelling study. After 24 h, the swollen adsorbent was filtered, and tissue paper was used to remove excess water. Then, the adsorbent was weighted (*m*_e_), and the swelling percentage was computed using eqn (1), as follows:1
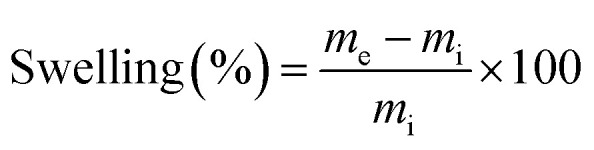


### 2.6. MB adsorption studies

To optimize the adsorption process, the influence of the MB sorption parameters such as adsorbent dose (0.5–4 g L^−1^), pH (2–10), initial MB concentration (10–50 mg L^−1^), sorption time (50–120 min), and temperature (25–50 °C) on the removal performance of the Hyd/WS, Hyd/WS/CoFe_2_O_4_, and Hyd/WS/CoFe_2_O_4_/ZIF-67 adsorbents was assessed in a batch mode. 0.1 M HCl or NaOH solution was used to adjust the pH of the MB solution. After the sorption period was over, the adsorbents were separated from the solution using a centrifuge and a magnet. The residual concentration of MB was measured with a UV-visible spectrophotometer (Shimadzu-1800, Japan) at *λ*_max_ = 664 nm. The removal percentage (*R*, %) and equilibrium adsorption capacity (*q*_e_) were computed using the following equations:2
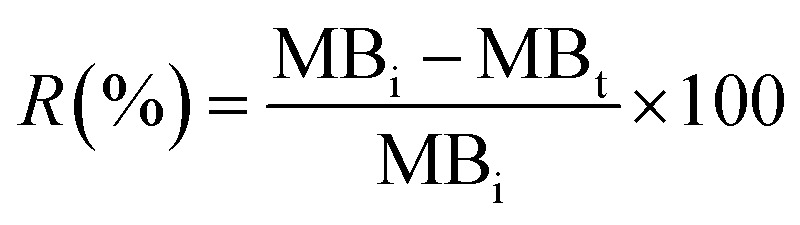
3
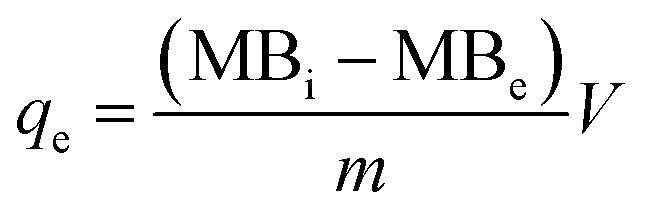
where MB_i_ (mg L^−1^), MB_e_ (mg L^−1^), *V*(mL), and *m*(g) denote the initial and equilibrium MB concentration, volume of MB solution, and mass of adsorbent, respectively.

### 2.7. Stability and reusability of synthesized adsorbent samples study

To evaluate the reusability of the adsorbents after the MB removal process, the adsorption efficiency was investigated up to 6 cycles. In each cycle, the adsorbent was isolated from the treated solution, added to 0.1 M nitric acid solution, and agitated for 3 h. After this time, the adsorbent was separated and washed using distilled water until it reached a neutral pH. The regenerated adsorbent was reused in the adsorption process.

## 3. Results and discussion

### 3.1. Characterization

**Fig. 2:**
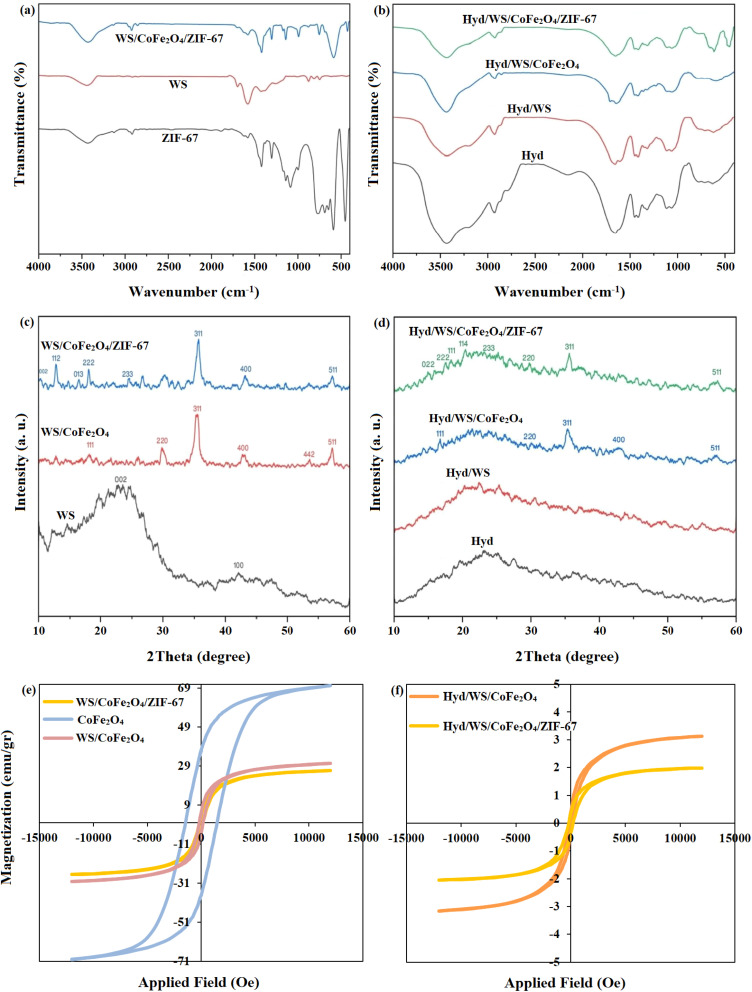
(a and b) FTIR, (c and d) XRD, and (e and f) VSM analyses of ZIF-67, WS, WS/CoFe_2_O_4_, WS/CoFe_2_O_4_/ZIF-67, Hyd, Hyd/WS, Hyd/WS/CoFe_2_O_4_, and Hyd/WS/CoFe_2_O_4_/ZIF-67 nanocomposite samples.

The outcomes of the FTIR analysis of the WS, ZIF-67, and WS/CoFe_2_O_4_/ZIF-67 samples are presented in Fig. 2a. In the FTIR spectrum of the WS sample, the peaks located at the wavenumbers of 3440, 2917, and 1580 cm^−1^ are related to the –OH, C–H and C=C bands, respectively.^22^ In the spectrum of the ZIF-67 sample, the peaks at the wavenumbers of 3433, 2929, 1451, 1320, and 1063 cm^−1^ are related to the N–H, C–H, C=O, N–H, and C–O bands, respectively.^23^ In the FTIR spectrum of WS/CoFe_2_O_4_/ZIF-67, the observed peaks at the wavenumbers of 3423, 2924, 1420, 1579, 586, and 425 cm^−1^ are assigned to the overlapping of the N–H and OH, C–H, C=N, C=N, Fe–O, and Co–N bands, respectively.^23,24^ In this sample, the intensity and position of the peaks related to WS and ZIF-67 were altered slightly, showing the high interaction between these components. The FTIR spectra of Hyd, Hyd/WS, Hyd/WS/CoFe_2_O_4_, and Hyd/WS/CoFe_2_O_4_/ZIF-67 are presented in Fig. 2b. In the FTIR spectrum of the Hyd sample, the observed peaks at the wavenumbers of 3433, 2929, 1451, 1320 and 1063 cm^−1^ are attributed to the stretching vibrations of the O–H & N–H, C–H, C=O, C–O and C–O–C bands, respectively.^25^ In the FTIR spectra of the Hyd/WS and Hyd/WS/CoFe_2_O_4_ samples, the intensity of the O–H & N–H, C=O, and C–O bands changed, demonstrating the high interaction of WS and WS/CeFe_2_O_4_ with the hydrogel matrix. In the FTIR spectrum of Hyd/WS/CoFe_2_O_4_/ZIF-67, all the peaks of Hyd, WS, and WS/CoFe_2_O_4_ were observed, and the observed peak at the wavenumber of 497 cm^−1^ is assigned to the Co–N band, showing the presence of ZIF-67 in the nanocomposite sample.

Fig. 2c shows the results of the XRD analysis of the ZIF-67, WS, WS/CoFe_2_O_4_, and WS/CoFe_2_O_4_/ZIF-67 samples. The ZIF-67 peaks in the 2θ range of 5° to 60° are located at 7.34°, 10.08°, 12.42°, 14.4°, 16.14°, 17.72°, 21.86°, 24.18° and 26.34°, which are assigned to the crystal planes of (011), (002), (112), (022), (013), (222), (114), (233) and (134), respectively, indicating the crystalline state of this sample. In the case of the WS sample, its XRD peaks are located at the 2*θ* values of 23.54° and 42.14°, which, according to the standard JCPDS Card No. 22-1086, correspond to the (002) and (111) crystal planes, respectively, indicating that this sample is amorphous. After the modification of WS with magnetic CoFe_2_O_4_ nanoparticles, peaks corresponding to the WS/CoFe_2_O_4_ sample were observed at the 2*θ* values of 18.02°, 30.38°, 35.7°, 43.28°, 53.56° and 57.32°, which correspond to the (111), (220), (311), (400), (442) and (511) crystal planes, respectively. After modifying WS/CoFe_2_O_4_ with ZIF-67, peaks were observed at 2θ values of 12.42°, 16.14°, 17.72°, 24.18°, 35.7°, 43.28°, and 57.26°, which correspond to the (112), (013), (222), (233), (311), (400), and (511) crystal planes, respectively, indicating the crystalline state of this sample.

The XRD patterns of Hyd, Hyd/WS, Hyd/WS/CoFe_2_O_4_, and Hyd/WS/CoFe_2_O_4_/ZIF-67 are presented in Fig. 2d. In the XRD pattern of Hyd, a broad peak appeared at 2*θ* = 23°, showing the semi-crystalline structure of this sample. In the XRD pattern of Hyd/WS, the characteristic peak of Hyd and WS overlapped at 2*θ* = 23°. In the XRD pattern of Hyd/WS/CoFe_2_O_4_, some sharp peaks appeared at the 2*θ* values of 16.72°, 30°, 35.4°, 47.1°, and 57.2°, which correspond to the (111), (220), (311), (400) and (511) crystal planes of the CoFe_2_O_4_ nanoparticles, respectively. In the XRD pattern of Hyd/WS/CoFe_2_O_4_/ZIF-67, the characteristic peaks of CoFe_2_O_4_ and ZIF-67 nanoparticles were observed, showing the successful integration of these nanoparticles in the hydrogel matrix.

The magnetic characteristic of adsorbents is a key property in the adsorption process, enabling the easy removal of the adsorbents through the application of a magnetic field. Thus, VSM analysis was performed to assess the magnetic properties of the CoFe_2_O_4_, WS/CoFe_2_O_4_/ZIF-67, Hyd/WS/CoFe_2_O_4_, and Hyd/WS/CoFe_2_O_4_/ZIF-67 samples, and the outcomes are presented in Fig. 2e and f. The magnetic saturation of CoFe_2_O_4_, WS/CoFe_2_O_4_, WS/CoFe_2_O_4_/ZIF-67, Hyd/WS/CoFe_2_O_4_, and Hyd/WS/CoFe_2_O_4_/ZIF-67 was determined to be 70.26, 30.26, 26.65, 3.12, and 1.98 emu g^−1^, respectively.^26,27^ The decrease in the magnetic saturation value of the hydrogels compared to the above-mentioned samples is due to the presence of non-magnetic components, including polymeric chains, in the structure of the hydrogels.^28^ However, although the magnetic saturation of the hydrogels decreased compared to the CoFe_2_O_4_, WS/CoFe_2_O_4_, and WS/CoFe_2_O_4_/ZIF-67 samples, their magnetic properties were still enough to separate them from aqueous solutions.

**Fig. 3:**
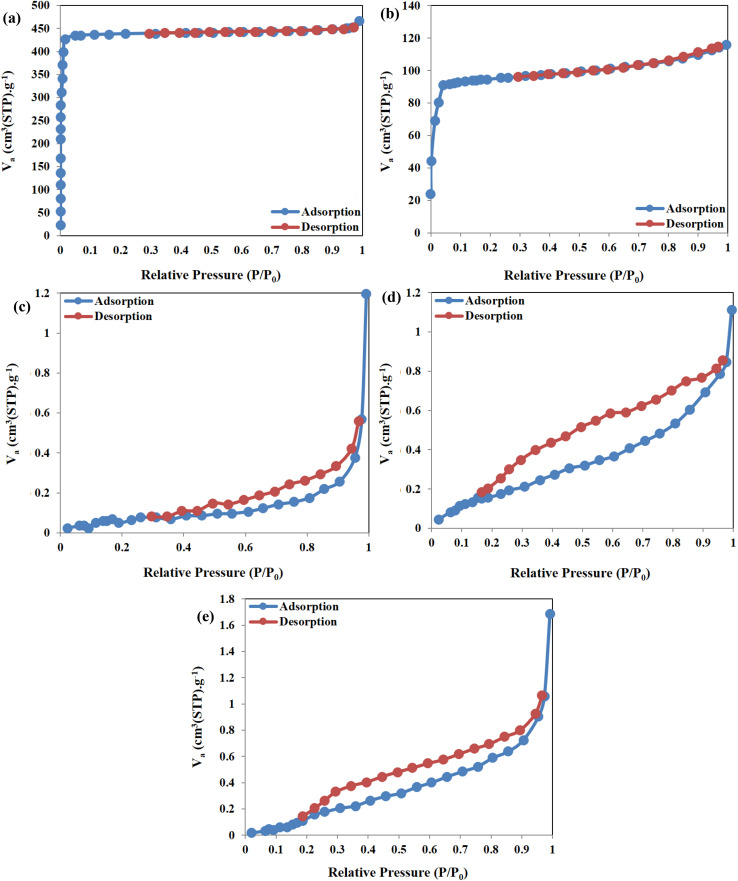
N_2_ ad(de)sorption isotherms of (a) ZIF-67, (b) WS/CoFe_2_O_4_/ZIF-67, (c) Hyd, (d) Hyd/WS/CoFe_2_O_4_ and (e) Hyd/WS/CoFe_2_O_4_/ZIF-67 nanocomposite samples.

BET is an efficient analysis for determining the specific active surface area, pore volume, and pore size. The N_2_ ad(de)sorption isotherm curves of ZIF-67, WS/CoFe_2_O_4_/ZIF-67, Hyd, Hyd/WS/CoFe_2_O_4_, and Hyd/WS/CoFe_2_O_4_/ZIF-67 are presented in Fig. 3a–e. The isotherm curves of ZIF-67 and WS/CoFe_2_O_4_/ZIF-67 are classified as type I, showing the presence of micropores in their structure. The isotherm curves of Hyd, Hyd/WS/CoFe_2_O_4_, and Hyd/WS/CoFe_2_O_4_/ZIF-67 belong to the IV type, demonstrating their mesoporous structure. The textural properties of the samples are tabulated in Table 1. The specific surface area of ZIF-67, WS/CoFe_2_O_4_/ZIF-67, Hyd, Hyd/WS/CoFe_2_O_4_, and Hyd/WS/CoFe_2_O_4_/ZIF-67 were determined to be 1837.4, 416.11, 0.3412, 0.5946, and 2.295 m^2^ g^−1^, respectively. The integration of WS/CoFe_2_O_4_ and WS/CoFe_2_O_4_/ZIF-67 into the hydrogel structure led to an increase in specific surface area because the distance between the polymer chains in the hydrogel structure was increased by the embedded nanoparticles. The average pore diameter of ZIF-67, WS/CoFe_2_O_4_/ZIF-67, Hyd, Hyd/WS/CoFe_2_O_4_, and Hyd/WS/CoFe_2_O_4_/ZIF-67 was computed to be 1.571, 1.7191, 19.44, 10.9 and 2.283 nm, respectively. According to the average pore diameter results, the prepared hydrogel adsorbents are materials with a mesoporous structure according to the IUPAC standard.

**Table 1:** Textural features of ZIF-67, WS/CoFe_2_O_4_/ZIF-67, Hyd, Hyd/WS/CoFe_2_O_4_, and Hyd/WS/CoFe_2_O_4_/ZIF-67 nanocomposite samples

Sample	BET surface area (m^2^ g^−1^)	Total pore volume (cm^3^ g^−1^)	Average pore size (nm)
ZIF-67	1837.4	0.7217	1.571
WS/CoFe_2_O_4_/ZIF-67	416.11	0.1788	1.7191
Hyd	0.34121	0.0016205	19.441
Hyd/WS/CoFe_2_O_4_	0.59468	0.0016583	10.9
Hyd/WS/CoFe_2_O_4_/ZIF-67	2.2295	0.0024841	2.283

**Fig. 4:**
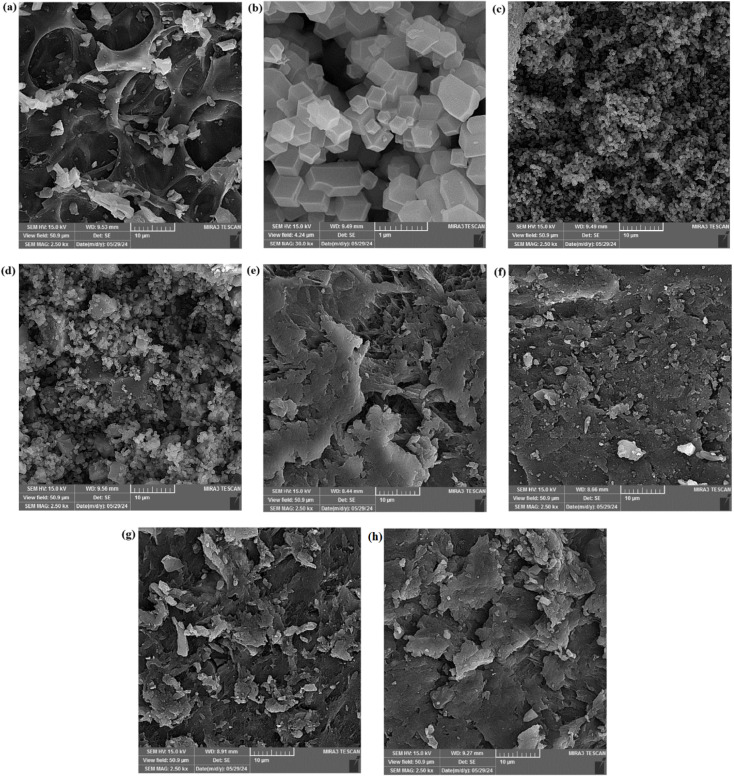
SEM images of (a) WS, (b and c) ZIF-67, (d) WS/CoFe_2_O_4_/ZIF-67, (e) Hyd, (f) Hyd/WS, (g) Hyd/WS/CoFe_2_O_4_ and (h) Hyd/WS/CoFe_2_O_4_/ZIF-67 nanocomposite samples.

The SEM images of WS, ZIF-67, WS/CoFe_2_O_4_/ZIF-67, Hyd, Hyd/WS/CoFe_2_O_4_, and Hyd/WS/CoFe_2_O_4_/ZIF-67 are demonstrated in Fig. 4. As presented in Fig. 4a, WS had a rough and porous structure with cavities of different sizes and shapes. These cavities facilitated the penetration of dye molecules into the adsorbent structure. Fig. 4b and c display the SEM images of the typical regular rhombic dodecahedron crystal structure of ZIF-67 at different magnifications. After integrating CoFe_2_O_4_ and ZIF-67, the structure of WS changed considerably. These nanoparticles filled the WS pores, and their surface got rougher. In the image of this sample in Fig. 4d, spherical and hexagonal particles indicate the presence of CoFe_2_O_4_ and ZIF-67 nanoparticles, respectively. As demonstrated in Fig. 4e, a rough surface and some interconnected pores were visible in the structure of Hyd, which are crucial for swelling and adsorption properties. According to Fig. 4f–h, the loose surface of Hyd got compact after integration with the WS, WS/CoFe_2_O_4_, and WS/CoFe_2_O_4_/ZIF-67 nanoparticles, enhancing the mechanical strength. Also, the surface of the nanocomposite hydrogel samples was flaky, unlike the hydrogel sample, especially in the Hyd/WS/CoFe_2_O_4_/ZIF-67 sample.

### 3.2. Impact of filler content on swelling and removal performance of nanocomposite hydrogels

**Fig. 5:**
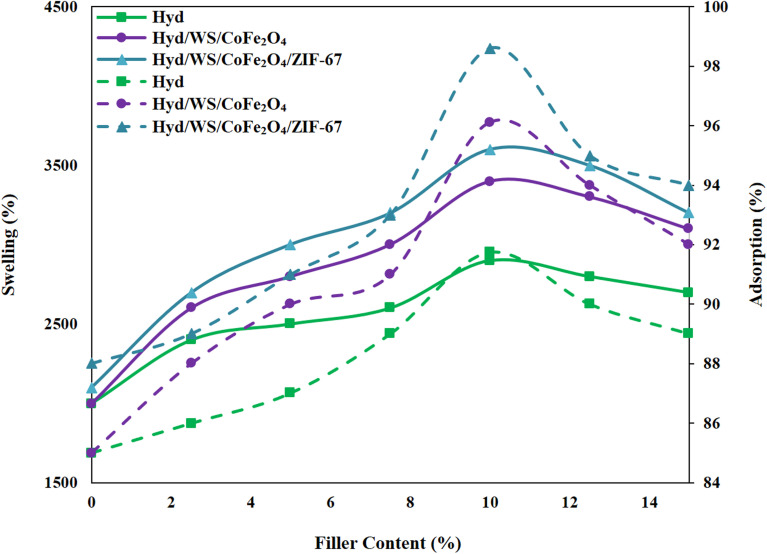
Impact of filler content on the swelling and removal performance of Hyd/WS, Hyd/WS/CoFe_2_O_4_ and Hyd/WS/CoFe_2_O_4_/ZIF-67 nanocomposite samples.

To optimize the swelling and removal performance, different weight percentages of WS/CoFe_2_O_4_ and WS/CoFe_2_O_4_/ZIF-67 nanoparticles (0–15 wt%) were embedded in the hydrogel matrix. The outcomes in Fig. 5 demonstrated that the swelling of Hyd increased from 2000% to 2950%, 3400%, and 3600% upon integration with 10 wt% of WS/CoFe_2_O_4_, and WS/CoFe_2_O_4_/ZIF-67 nanoparticles, respectively, then it decreased. Also, the removal performance of Hyd was elevated from 85.74% to 91.74%, 96.11%, and 98.61% by integration with 10 wt% of WS, WS/CoFe_2_O_4_, WS/CoFe_2_O_4_, and WS/CoFe_2_O_4_/ZIF-67 nanoparticles, respectively. The increased swelling and removal performance of Hyd could be due to its increase in functional groups such as carboxyl and hydroxyl and specific surface area.^29^ By further integrating these nanoparticles at 10 wt%, these parameters decreased, which is related to the agglomeration of these nanoparticles, leading to the filling of the pores of the adsorbents, and thus the MB molecules could not diffuse into the adsorbent structure and interact with the adsorption sites. Nanocomposite hydrogels with 10 wt% of filler content were selected for further experiments.

### 3.3. Investigation of adsorption parameters on elimination of MB

**Fig. 6:**
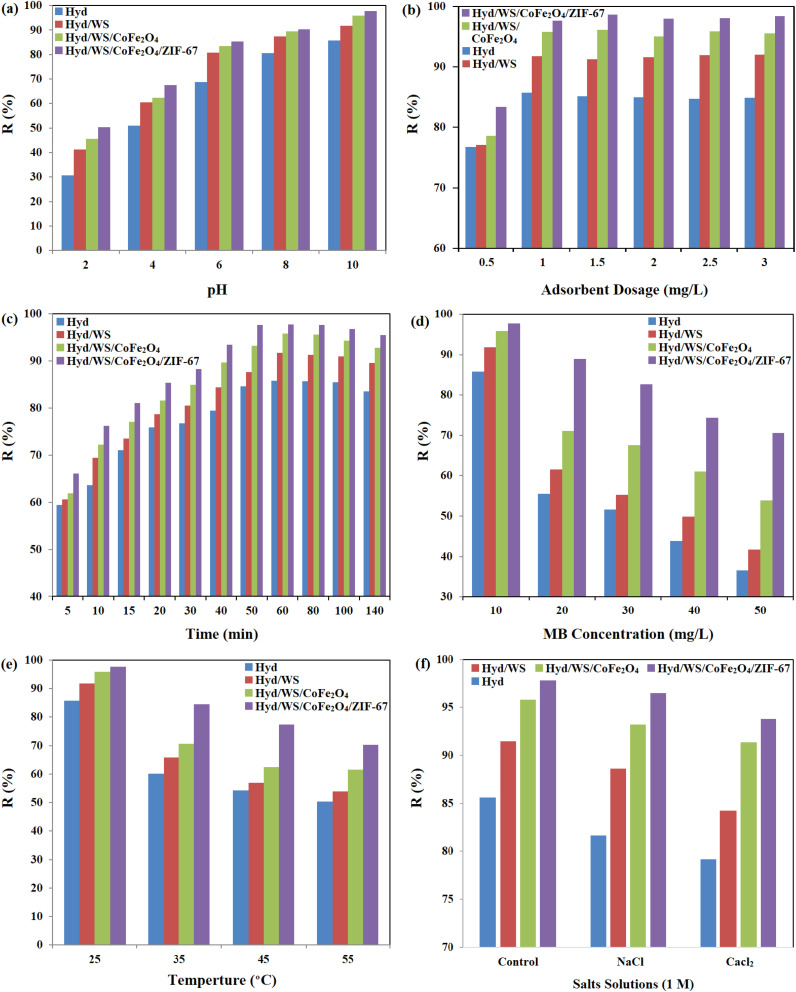
Impact of (a) pH, (b) adsorbent dose, (c) contact time, (d) MB initial concentration, (e) temperature, and (f) counter ions on the removal performance of Hyd, Hyd/WS, Hyd/WS/CoFe_2_O_4_ and Hyd/WS/CoFe_2_O_4_/ZIF-67 nanocomposite samples.

The impact of pH on the removal performance of the Hyd, Hyd/WS, Hyd/WS/CoFe_2_O_4_, and Hyd/WS/CoFe_2_O_4_/ZIF-67 nanocomposite adsorbents was assessed in the range of 2–10. The outcomes in Fig. 6a showed that the removal performance of the Hyd, Hyd/WS, Hyd/WS/CoFe_2_O_4_, and Hyd/WS/CoFe_2_O_4_/ZIF-67 nanocomposite adsorbents was enhanced from 30.74%, 41.26%, 45.64%, and 50.37% to 85.74%, 91.74%, 95.83% and 97.72% with an increment in pH from 2 to 10. With an increment in pH, the functional groups in the adsorbents, including –COOH and –Fe–OH, were ionized to –COO– and –Fe–O–, and thus the cationic MB molecules could interact with the adsorbents through electrostatic interactions. Another reason for the low removal performance in an acidic medium is the presence of H^+^ ions in the pollutant medium, which competes with MB molecules to interact with the adsorption sites.^30^

Given that the adsorption process involves interaction between the adsorbent and the adsorbate, the adsorbent dose should be investigated in this process. The impact of the adsorbent dose in the range of 0.5 to 3 g L^−1^ was investigated, and the results are presented in Fig. 6b. The results showed that the removal performance was elevated from 76.79%, 77.08%, 78.63%, and 83.34% to 85.74%, 91.74%, 95.83%, and 97.72% with an increment in the adsorbent dose from 0.5 to 1 g L^−1^. With an increment in adsorbent, the number of adsorption sites and surface area to interact with MB molecules increased, and the removal performance was enhanced. However, the saturation of the adsorption sites and agglomeration of the adsorbent particles lead to a decrement in surface area, and the removal performance became constant with a further increase in adsorbent dose from 1 g L^−1^.^31^ Thus, the optimum adsorbent dose for the synthesized adsorbents was selected as 1 g L^−1^.

The influence of contact time on the removal performance of Hyd, Hyd/WS, Hyd/WS/CoFe_2_O_4_, and Hyd/WS/CoFe_2_O_4_/ZIF-67 was investigated in the range of 5 to 140 min. As demonstrated in Fig. 6c, the adsorption process reached equilibrium in the first 50 min. During the initial period of the adsorption process, the majority of the adsorption sites were unoccupied and prepared to interact with MB molecules, and thus the removal performance increased with time.^32^ With the saturation of the adsorption sites and filling of adsorbent pores with MB molecules, the removal performance reached equilibrium and the removal performance stabilized.

The impact of MB concentration on the removal performance of Hyd, Hyd/WS, Hyd/WS/CoFe_2_O_4_, and Hyd/WS/CoFe_2_O_4_/ZIF-67 was investigated, and the outcomes are presented in Fig. 6d. With an increase in dye concentration from 10 to 50 mg L^−1^, the removal performance of the adsorbents decreased. This can be explained by the limited availability of active sites on the adsorbents and the electrostatic repulsion between the MB molecules that are adsorbed on the surface and those in the aqueous solution.^33^

The impact of temperature on the removal performance of Hyd, Hyd/WS, Hyd/WS/CoFe_2_O_4_, and Hyd/WS/CoFe2O4/ZIF-67 was investigated, and the results are depicted in Fig. 6e. With an increment in temperature from 25 °C to 55 °C, the removal performance decreased from 85.74%, 91.74%, 95.83%, and 97.72% to 50.27%, 53.98%, 61.56%, and 70.28% for Hyd, Hyd/WS, Hyd/WS/CoFe_2_O_4_, and Hyd/WS/CoFe_2_O_4_/ZIF-67, respectively. The reduction in removal performance showed that the decontamination process is exothermic.^34^ The decrement in removal performance can be related to the weakening of the electrostatic interactions and hydrogen bonds between the adsorbents and the MB molecules.

The presence of counter ions can significantly influence adsorption, particularly in systems involving charged adsorbents and adsorbates. The present study investigated the effect of Na^+^ and Ca^2+^ ions at a concentration of 1 M on the removal efficiency by Hyd, Hyd/WS, Hyd/WS/CoFe_2_O_4_, and Hyd/WS/CoFe_2_O_4_/ZIF-67. Based on the results in Fig. 6f, the presence of these cationic ions in the pollutant solution led to a decrement in the removal efficiency of the adsorbents. Na^+^ and Ca^2+^ ions compete with MB molecules to interact with the binding sites on the adsorbent. The reduction in the removal efficiency of the adsorbents in the presence of Ca^2+^ ions was higher than Na^+^ ions because Ca^2+^ ions can act as a crosslinker in the adsorbent structure and limit the diffusion of MB molecules. Also, Ca^2+^ ions are smaller than Na^+^ ions, and thus they can easily penetrate the structure of the adsorbents.

### 3.4. Kinetic study

Kinetic studies on the adsorption process are essential for understanding the mechanism controlling the adsorbent and adsorbate interactions. Four common kinetic models, including pseudo-first-order (PFO, eqn (4)), pseudo-second-order (PSO, eqn (5)), Elovich (eqn (6)), and intra-particle diffusion (IPD, eqn (7)) models, were used for the kinetic study.

4 *q*_t_ = *q*_e_(1 − exp(−*k*_1_*t*))

5
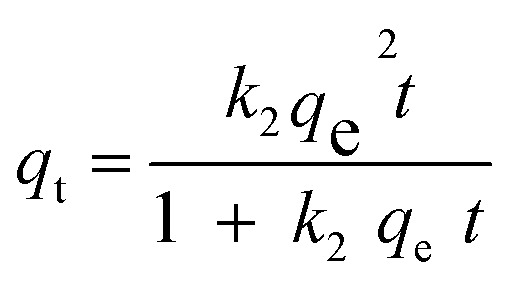
6
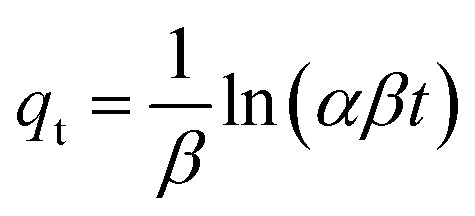

7 *q*_t_ = *k*_i,d_*t*^0.5^ + *C*_i_

**Fig. 7:**
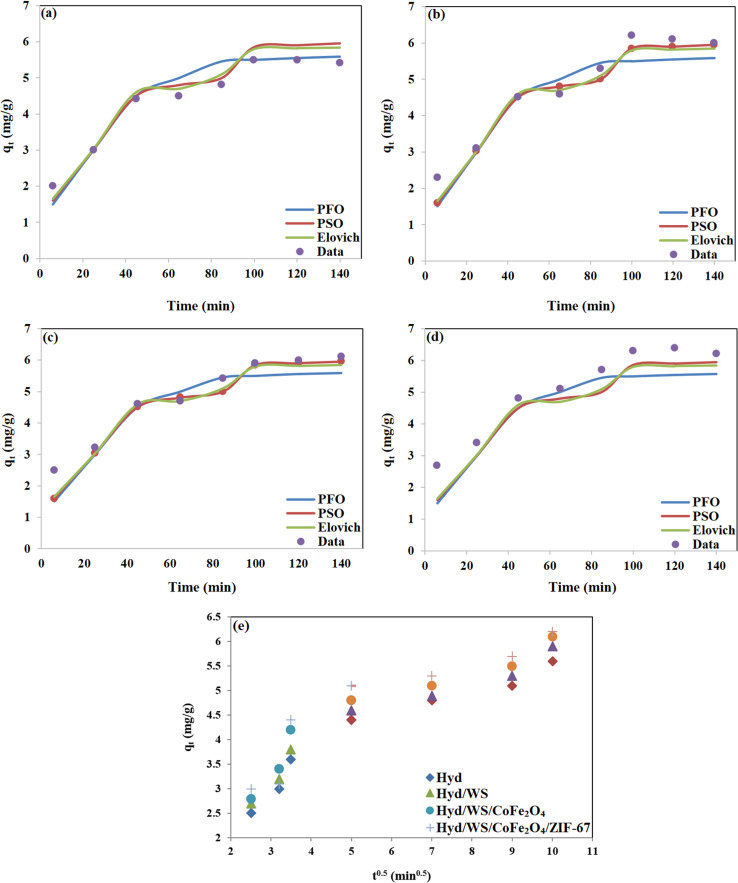
Non-linear regression of kinetic models for (a) Hyd, (b) Hyd/WS, (c) Hyd/WS/CoFe_2_O_4_, (d) Hyd/WS/CoFe_2_O_4_/ZIF-67 nanocomposite samples and (e) intra-particle diffusion model for these samples.

The regression results are depicted in Fig. 7a–e and the regression parameters and constants are tabulated in Table 2. The results showed that the experimental kinetic data for all the synthesized adsorbents were best fitted with PSO, and thus the rate of the MB decontamination process is mainly controlled by chemisorption.^35^ Based on the regression results using the intra-particle model, the MB adsorption process occurred in two stages. In the first one, MB molecules diffused from the aqueous solution to the adsorbent surface, and in the second one, they diffused through the pores of the adsorbents. The computed rate constant for the first stage was higher than the second one, and thus the intra-particle diffusion step mostly controlled the rate of the MB decontamination process.

**Table 2:** Kinetic parameters for Hyd, Hyd/WS, Hyd/WS/CoFe_2_O_4_, and Hyd/WS/CoFe_2_O_4_/ZIF-67 nanocomposite samples^a^

Parameter	Hyd	Hyd/WS	Hyd/WS/CoFe_2_O_4_	Hyd/WS/CoFe_2_O_4_/ZIF-67
**Pseudo-first-order model**				
*q*_e.cal_ (mg g^−1^)	3.162	3.781	4.12	4.16
*k*_1_ (L min^−1^)	0.0261	0.0334	0.0390	0.0401
*R* ^2^	0.954	0.972	0.965	0.970

**Pseudo-second-order model**				
*q*_e.cal_ (mg g^−1^)	4.723	5.172	5.327	5.401
*k*_2_ (L min^−1^)	0.0048	0.00721	0.00763	0.0077
*R* ^2^	0.962	0.978	0.983	0.976

**Elovich model**				
*α* (mg g^−1^ min^1/2^)	0.239	0.373	0.402	0.452
*β* (g mg^−1^)	0.928	0.901	0.896	0.911
*R* ^2^	0.938	0.943	0.959	0.961

**Intra-particle diffusion model**				
*K*_IPD1_ (mg g^−1^ min^1/2^)	0.7583	0.7583	0.7868	0.8829
*C*_i1_ (mg g^−1^)	0.6832	0.8832	1.00	0.7658
*R* ^2^	0.9537	0.9537	0.8885	0.8266
K_IPD2_ (mg g^−1^ min^1/2^)	0.222	0.2424	0.2424	0.2085
*C*_i2_ (mg g^−1^)	3.2542	3.2966	3.4966	3.9593
*R* ^2^	0.9474	0.9145	0.9145	0.9061

a *q*_e_: calculated equilibrium adsorption capacity, *k*_1_ (min^−1^): rate constant of PSF, *k*_2_ (g mg^−1^.min): rate constant of PSO, *K*_i,d,_: IPD rate constant, and *C*_i_: a constant related to the boundary layer.

### 3.5. Isotherm study

To examine the most suitable isotherm model, four types of common isotherms, including Langmuir (eqn (8)), Freundlich (eqn (9)), Dubinin–Radushkevich (D–R) (eqn (10)), and Temkin (eqn (11)), were evaluated, as follows:8
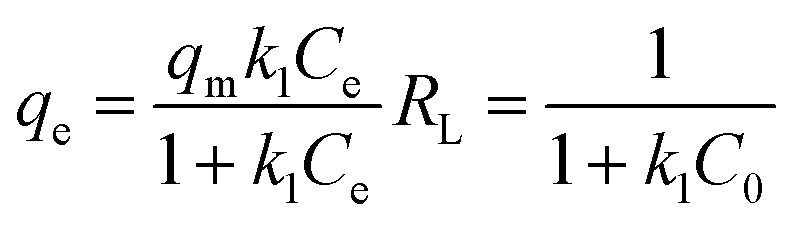

9 *q*_e_ = *K*_F_*C*^1/*n*^_e_

10
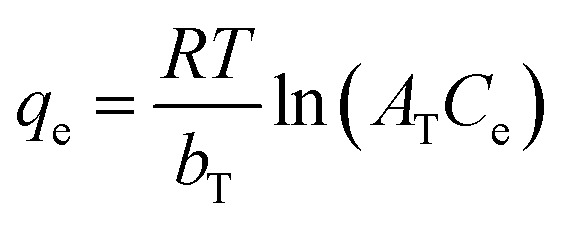
11
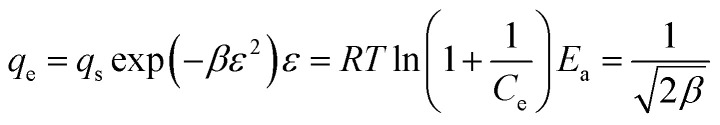


**Fig. 8:**
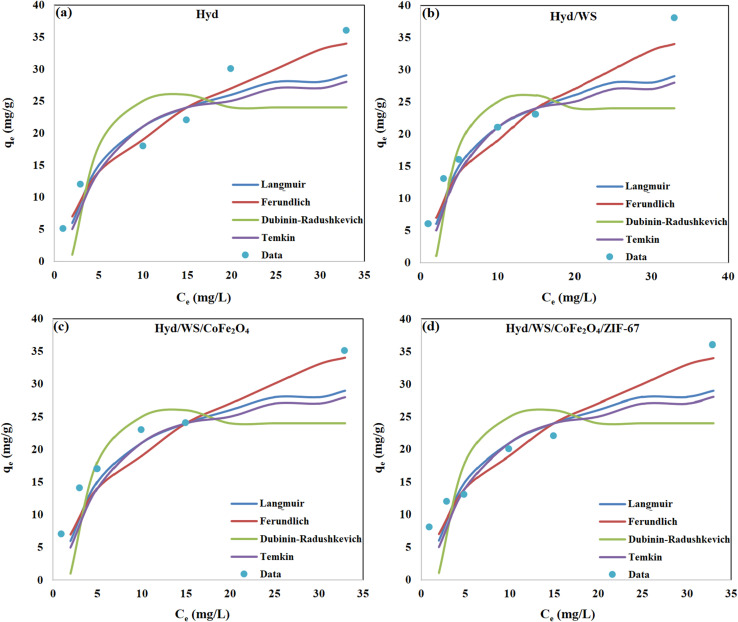
Non-linear regression of isotherm models for (a) Hyd, (b) Hyd/WS, (c) Hyd/WS/CoFe_2_O_4_ and (d) Hyd/WS/CoFe_2_O_4_/ZIF-67 nanocomposite samples.

The fitted curves are demonstrated in Fig. 8a–d and the fitting parameters are listed in Table 3. The comparison of the correlation coefficients (*R*^2^) indicated that the Freundlich isotherm fit the equilibrium data of the Hyd, Hyd/WS, Hyd/WS/CoFe_2_O_4_, and Hyd/WS/CoFe_2_O_4_/ZIF-67 adsorbents better than the other three models. Hence, MB adsorption on the adsorbents is multilayer.^36^ The computed *n* value for all the adsorbents was in the range of 1–10, showing the favorability of the adsorption process.^37^ The effectiveness of the prepared adsorbents in extracting methylene blue (MB) from aqueous solutions aligns with findings reported in earlier research, as shown in Table 4. These results suggest that the developed adsorbents show promise for the removal of MB from contaminated water.

**Table 3:** Non-linear fitting parameters of isotherm models for MB adsorption by Hyd, Hyd/WS, Hyd/WS/CoFe_2_O_4_, and Hyd/WS/CoFe_2_O_4_/ZIF-67 nanocomposite samples^a^

Parameter	Hyd	Hyd/WS	Hyd/WS/CoFe_2_O_4_	Hyd/WS/CoFe_2_O_4_/ZIF-67
**Langmuir**				
*q*_m_ (mg g^−1^)	36.36	41.152	52.91	69.93
*K*_L_	0.164	0.173	0.215	0.323
*R*_L_	0.752–0.924	0.7428–0.92	0.698–0.902	0.607–0.86
*R* ^2^	0.857	0.886	0.898	0.8072

**Freundlich**				
*K*_F_ (mg g^−1^(L mg^−1^)^1/*n*^)	2.640	4.01	5.52	8.21
*n*	2.016	2.178	2.99	4.928
*R* ^2^	0.972	0.978	0.972	0.9805

**Dubinin-Radushkevich**				
*E* (kJ mol^−1^)	0.223	0.223	0.235	0.316
*q*_m_ (mg g^−1^)	41.90	47.02	58.98	75.64
*β* × 10^−6^ (mol^2^ J^−2^)	0.1	0.1	9	5
*R* ^2^	0.8727	0.8929	0.885	0.723

**Temkin**				
*b*_T_ (kJ mol^−1^)	0.085	0.083	0.10	0.14
*A*_T_ (L g^−1^)	1.005	1.005	1.006	1.008
*R* ^2^	0.956	0.959	0.962	0.975

a *q*_m_ (mg g^−1^): maximum adsorption capacity, *K*_L_ (L mg^−1^): Langmuir adsorption constant, *K*_F_ and *n*: Freundlich model constants, *b* (J mol^−1^) and *A*_T_ (L g^−1^): Temkin constants, *R*: universal constant of gases, *T*(*K*): absolute temperature, *ε*: Polanyi coefficient and *β* (mol g^−1^)^2^: activity coefficient.

**Table 4:** Comparison of the process of removing methylene blue dye by the adsorbents prepared in the present study with other adsorbents used in previous studies

Absorbent	*q*_m_ (mg g^−1^)	Percentage removal (%)	References
Hydrogel beads based on carboxymethyl cellulose (CMC), alginate (Alg) and graphene oxide (GO)	45.045	96.22	38
Sodium-alginate/acrylamide (Na-alginate/AAm) cross-linked hydrogel	78.1	90	39
*N*-isopropylacrylamide (NIPAAm)/itaconic acid (IA)/pumice composite hydrogel	22.62	70.8	40
*N*,*N*-dimethylacrylamide and 2-hydroxyethyl methacrylate copolymer (p (HEMA-*co*-DMAa))	80.27	>60	41
*N*-isopropylacrylamide (NIPAAm)/itaconic acid (IA)/pumice composite hydrogel	22.18	70.8	40
Starch/poly(acrylic acid) hydrogel	26.7	66.7	42
CMC-g-poly(AAm)	36.36	85.74	This work
CMC-g-poly(AAm)/WS	41.152	91.74	This work
CMC-g-poly(AAm)/WS/CoFe_2_O_4_	52.91	96.11	This work
CMC-g-poly(AAm)/WS/CoFe_2_O_4_/ZIF-67	69.93	98.61	This work

### 3.6. Thermodynamic study

Investigating the thermodynamics can offer a deeper understanding of the adsorption process. In this research, key thermodynamic parameters, including the change in Gibbs free energy (Δ*G*°), enthalpy change (Δ*H*°), and entropy change (Δ*S*°), were calculated using the following equations:12
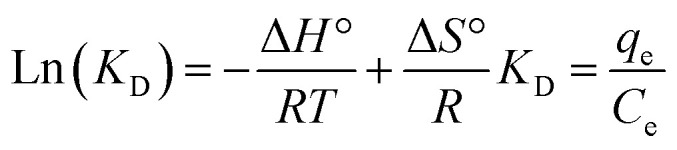

13 Δ*G*° = −*RT* ln(*K*_D_)

**Fig. 9:**
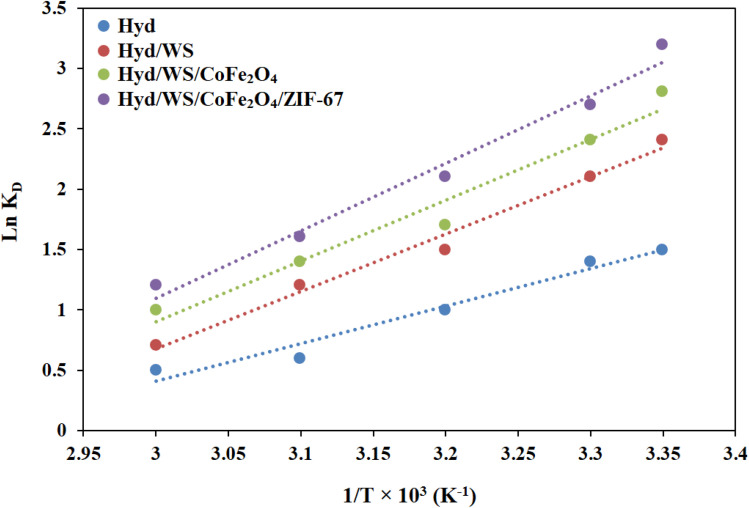
Linear relation between Ln(*K*_D_) and 1/*T* for the Hyd, Hyd/WS, Hyd/WS/CoFe_2_O_4_ and Hyd/WS/CoFe_2_O_4_/ZIF-67 nanocomposite samples.

The change in Gibbs free energy (Δ*G*°, in kJ mol^−1^) was computed using eqn (13). The change in standard enthalpy energy (Δ*H*°, in kJ mol^−1^) and the change in standard entropy energy (Δ*S*°, in kJ mol^−1^ K^−1^) were calculated by plotting Ln(*K*_D_) *vs.* 1/*T* (eqn (12)), as presented in Fig. 9. As described in Table 5, the value of Δ*G*° for the Hyd, Hyd/WS, Hyd/WS/CoFe_2_O_4_, and Hyd/WS/CoFe_2_O_4_/ZIF-67 adsorbents were negative, revealing that the MB decontamination process happened spontaneously. Also, the spontaneity of the MB removal process diminished as the temperature increased, indicating that higher temperatures are not suitable for the adsorption of MB molecules. The Δ*H*° values for the Hyd, Hyd/WS, Hyd/WS/CoFe_2_O_4_, and Hyd/WS/CoFe_2_O_4_/ZIF-67 adsorbents were calculated to be −31.071, −32.063, −33.154, and −38.67 kJ mol^−1^, respectively, showing the exothermic nature of the adsorption process. Also, the computed Δ*S*° values for the Hyd, Hyd/WS, Hyd/WS/CoFe_2_O_4_, and Hyd/WS/CoFe_2_O_4_/ZIF-67 adsorbents were −0.0848, −0.089, −0.099, and −0.103 kJ mol^−1^ K, respectively. The negative values of Δ*S*° showed that the random collisions of MB molecules and the surface of the synthesized adsorbents were reduced during the adsorption process.^43^

**Table 5:** Thermodynamic parameters associated with the MB decontamination process for the Hyd, Hyd/WS, Hyd/WS/CoFe_2_O_4_, and Hyd/WS/CoFe_2_O_4_/ZIF-67 nanocomposite samples

*T* (°C)	ΔG° (kJ mol^−1^)	Δ*H*° (kJ mol^−1^)	Δ*S*° (kJ (mol k)^−1^)
**Hyd**			
25	−5.78	−31.071	−0.0848
35	−4.93		
45	−4.08		
55	−3.24		

**Hyd/WS**			
25	−5.51	−32.063	−0.0890
35	−4.62		
45	−3.73		
55	−2.84		

**Hyd/WS/CoFe**_**2**_ **O**_**4**_			
25	−6.06	−33.154	−0.099
35	−5.15		
45	−4.24		
55	−3.34		

**Hyd/WS/CoFe**_**2**_ **O**_**4**_ **/ZIF-67**			
25	−7.67	−38.67	−0.104
35	−6.64		
45	−5.60		
55	−4.56		

### 3.7. Reusability study

**Fig. 10:**
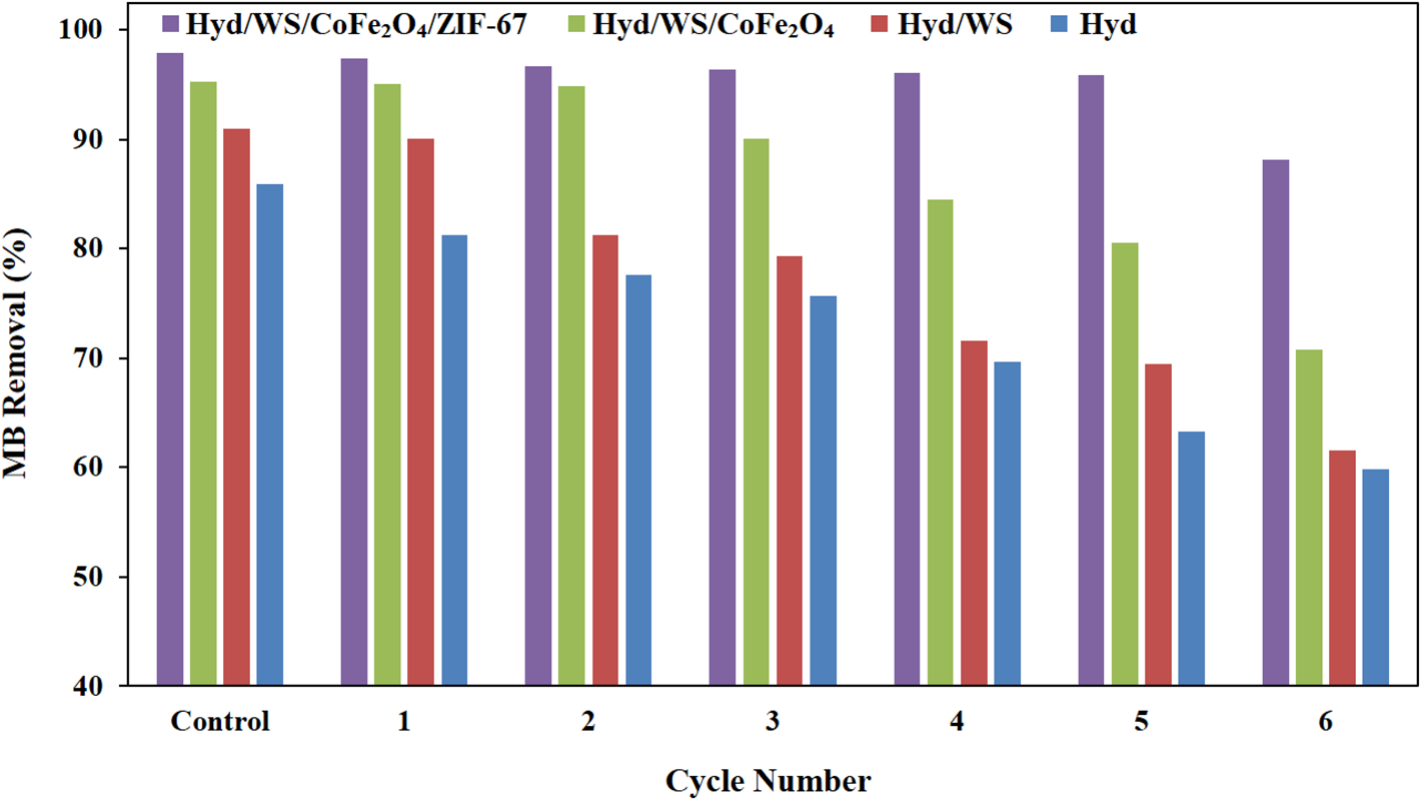
Reusability of Hyd, Hyd/WS, Hyd/WS/CoFe_2_O_4_ and Hyd/WS/CoFe_2_O_4_/ZIF-67 nanocomposite samples.

The reusability of the Hyd, Hyd/WS, Hyd/WS/CoFe_2_O_4_, and Hyd/WS/CoFe_2_O_4_/ZIF-67 adsorbents was assessed in 6 successive ad(de)sorption cycles, and results are depicted in Fig. 10. The synthesized adsorbents especially Hyd/WS/CoFe_2_O_4_/ZIF-67 showed a high reusability performance. The removal performance of the Hyd/WS, Hyd/WS/CoFe_2_O_4_, and Hyd/WS/CoFe_2_O_4_/ZIF-67 adsorbents was nearly constant for up to 2, 3, and 5 ad(de)sorption cycles, respectively, and then it decreased. The reduction in removal performance could be attributed to the blockage of the adsorption sites and the destruction of the adsorbent structure.

### 3.8. Adsorption mechanism

**Fig. 11:**
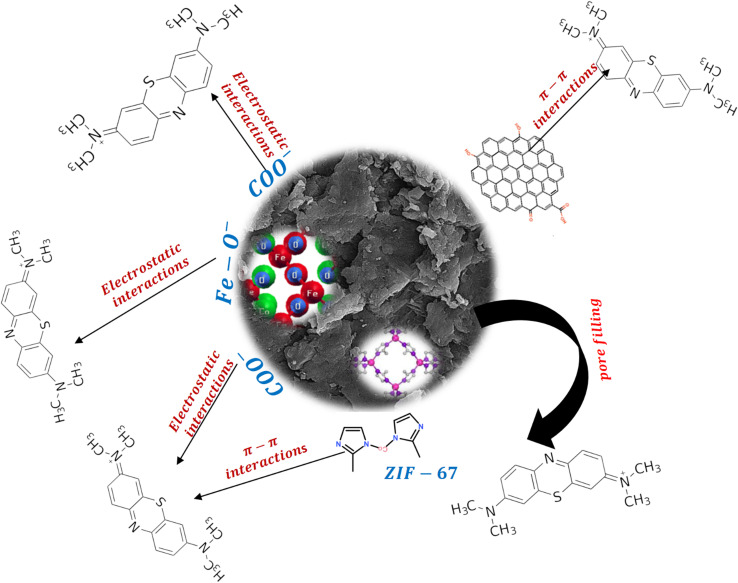
Scheme of MB adsorption mechanism by Hyd/WS/CoFe_2_O_4_/ZIF-67.

Depending on the chemical and textural features of adsorbents and the chemical structure of pollutants, the decontamination process can proceed *via* various mechanisms, including hydrogen bonding, electrostatic interactions, and π–π interactions. At the optimum pH of the elimination process, MB has a cationic nature, and the carboxyl groups of adsorbents were ionized to –COO^−^, and thus electrostatic interactions could occur between the adsorbents and MB molecules. Based on the SEM and BET analysis, the adsorbents had a porous structure, and thus the MB molecules could penetrate the adsorbent structure; hence, pore filling is another suggested mechanism. In addition to these mechanisms, π–π interactions could be formed between the aromatic rings of the MB molecules and WS and ZIF-67 of the adsorbents and hexagonal rings of CMC. Fig. 11 demonstrates a scheme of the MB adsorption mechanism by Hyd/WS/CoFe_2_O_4_/ZIF-67.

## 4. Conclusion

In the present study, a poly(AAm)-grafted CMC hydrogel was synthesized *via* the free radical polymerization method to eliminate MB from water media. To enhance its removal performance, biochar from walnut shells was integrated into the hydrogel matrix. The walnut shell biochar was decorated with CoFe_2_O_4_ and ZIF-67 nanoparticles and incorporated into the hydrogel matrix to enhance its removal performance further. The maximum removal performance of the adsorbents was obtained at pH = 10, adsorbent dose of 1 g L^−1^, contact time of 50 min, initial concentration of 10 mg L^−1^, and temperature of 25 °C. The kinetic and equilibrium data assessment showed the prominence of the pseudo-second-order and Freundlich models, respectively. Also, the thermodynamic study demonstrated that the removal of MB by the synthesized adsorbents was spontaneous and exothermic. Further, the reusability study demonstrated that Hyd/WS/CoFe_2_O_4_/ZIF-67 could be reused in six successive ad(de)sorption cycles.

## Author contributions

Seyed Jamaleddin Peighambardoust designed the study, interpreted the results, and revised the manuscript critically for important intellectual content; Somayyeh Rezaei-Aghdam performed the samples' synthesis and collected test data; and Parisa Mohammadzadeh Pakdel drafted and advised on the conceptualization of the manuscript, and Javaneh Sakhaei Niroumand advised on conceptualization; Mika Sillanpää revised, commented and edited the first draft of the manuscript. All authors approved the final version of the manuscript and agreed to be accountable for all the aspects of the work.

## Conflicts of interest

This research was conducted without commercial or financial relationships that could be construed as a potential conflict of interest. The authors, therefore, declare no conflict of interest.

## Data Availability

All data is included in the manuscript.
